# From social media engagement to public health action: the role of social media in sustainable malaria elimination

**DOI:** 10.3389/fpubh.2026.1987769

**Published:** 2026-10-30

**Authors:** Xun Zheng, Gelin Huang

**Affiliations:** 1 School of Media and Communication, Shanghai Jiao Tong University, Shanghai, China; 2 School of Humanities, Wenzhou University, Wenzhou, China

**Keywords:** community engagement, malaria elimination, public health, social media, surveillance systems, vector control

## Abstract

**Background:**

Malaria remains a major global public health challenge, for which conventional malaria prevention and elimination depend on malaria-related knowledge, perceptions, behaviors, and public responses, but in reality, they require integrated surveillance, community engagement, behavioral change, and the rapid translation of evidence into context-specific public health action. Social media has emerged as an important module involving communities in intervention design and implementation, but evidence remains fragmented across measurable malaria outcomes and analytics.

**Objective:**

To map and synthesize the available evidence using social media for the malaria elimination continuum, limitations, and gaps, and to inform the integration of social media intelligence into malaria programs and policy.

**Methodology:**

A scoping review was conducted in accordance with PRISMA-ScR using a predefined search and screening framework. Studies were eligible if they examined the use or evaluation of social media platforms or social media-derived data. Records were screened for eligibility, and data were charted according to study characteristics, geographical setting, platform, social media function, malaria application, and demonstrated public health action level. Findings were synthesized descriptively and mapped along an engagement-to-action pathway.

**Results:**

Thirty studies published between 2016 and 2026 were included. Eighteen originated from African settings, eight were global/international, three were from China, and one was from India. Twitter/X and Facebook were the most frequently represented platforms. Health education and behavior-change communication was the most common primary social-media role (33.3%), while misinformation and risk communication accounted for 23.3%. At the highest demonstrated public-health action level, 10 studies reported reach, engagement, or knowledge outcomes; 12 generated public-health intelligence; and two reported malaria-related health outcomes.

**Conclusion:**

Social media has evolved from disseminating malaria information to supporting social listening and program response, but a substantial engagement-to-action gap remains, with limited evidence linking social media activities to malaria-related health outcomes. Therefore, social media should be integrated as a complementary component of surveillance rather than considered a stand-alone elimination intervention.

## 1 Background

Malaria remains a major global public health challenge as one of the most persistent infectious diseases. Despite substantial advances in diagnosis, prevention, treatment, and vector control, an estimated 282 million malaria cases and 610,000 deaths occurred globally in 2024, with the highest burden concentrated in sub-Saharan Africa (1). Although several countries have achieved or are approaching elimination, progress remains uneven and vulnerable to persistent transmission, population mobility, health-system constraints, antimalarial drug and insecticide resistance, geographic expansion, climate variability, and declining intervention coverage (1, 2). These challenges underscore the need to shift from conventional disease-control approaches to adaptive strategies that can sustain gains and respond rapidly to evolving transmission patterns.

Contemporary malaria prevention and elimination increasingly depend on the interaction between effective interventions, surveillance, sustained vector-control coverage, community participation and acceptance of emerging malaria vaccines and health-system capacity to rapidly translate evidence into context-specific action (3). Conventional epidemiological, diagnostic, and entomological surveillance provides essential information on where and when malaria transmission occurs (4). However, these systems provide limited insight into health-seeking intentions, community perceptions, behavioral barriers, intervention acceptance, misinformation, and emerging concerns that influence program implementation. A recent scoping review of community engagement approaches in malaria-endemic settings demonstrated that community involvement can support malaria prevention, control, and elimination, but also highlighted that existing interventions have disproportionately emphasized informing communities, while consultation, collaboration, and co-leadership remain comparatively underdeveloped (5).

Social media has emerged as an important component involving communities in intervention design and implementation. Platforms such as Instagram, Facebook, TikTok, WhatsApp, YouTube, X, and other communities facilitate the rapid dissemination and exchange of health-related information across geographic and social boundaries (6). Unlike traditional one-directional health communication, social media provides two-way communication, creating opportunities for malaria programs to move beyond conventional message dissemination toward community engagement, social listening, and real-time identification of information needs (7).

Digital conversations provide signals about disease-related knowledge, attitudes, perceptions, acceptance of interventions, treatment-seeking behavior, vaccine confidence, and misinformation. When appropriately analyzed and validated, these signals complement conventional surveillance and help identify recurring concerns about specific diseases. However, at the same time, social media can accelerate the spread of misinformation and distrust, as in April 2026, WHO’s Special Programme for Research and Training in Tropical Diseases (TDR) highlighted research efforts specifically addressing social-media influencers spreading rumours about malaria vaccines, illustrating how online information environments are becoming part of the implementation context for malaria interventions (8). Thus, social media simultaneously functions as a mechanism for strengthening health communication and as an environment for both an opportunity as well as a threat.

However, the malaria-specific evidence remains fragmented across social media applications. Existing studies also vary considerably in their platforms, populations, analytical methods, and outcome measures, making it difficult to determine whether online engagement translates into meaningful programmatic, behavioral, or epidemiological outcomes. A comprehensive mapping of this evidence is therefore needed to clarify what social media can currently contribute to malaria elimination, where the evidence is strongest, and where important methodological and implementation gaps remain. Therefore, this scoping review aims to map and characterize the available evidence on the use of social media across the malaria elimination continuum, including its applications in social media surveillance and community engagement. Further characterization of the populations, settings, analytical methods, and outcomes was conducted, and methodological, ethical, equity, and implementation gaps that should guide future research and malaria-program development were identified.

## 2 The changing landscape of malaria elimination

The transition from malaria control toward elimination changes the information and implementation requirements of malaria control and prevention programs. While control strategies primarily seek to reduce disease burden, elimination requires rapid investigation of cases and foci, precise identification of residual transmission, targeted deployment of interventions, and prevention of re-establishment (3, 9). Surveillance consequently becomes not only a monitoring mechanism but also an instrument for programmatic decision-making. At the same time, malaria elimination is strongly dependent on community behavior and participation. As transmission declines, maintaining risk perception and community commitment may become increasingly difficult, particularly where malaria is no longer perceived as an immediate threat (5).

This creates an important gap between what conventional surveillance can measure and what communities experience or communicate. Routine systems are limited to identifying cases, intervention coverage, and other program indicators and do not adequately capture reasons for intervention refusal or emerging concerns. Thus, social media offers a potential complementary source of community-level information by providing access to publicly expressed perceptions, discussions, questions, and reactions to malaria interventions, with its role better understood as broader than communication. Table 1 presents an author-derived conceptual synthesis of major information gaps relevant to malaria elimination and potential areas in which social-media-derived information could complement existing program systems. The information gaps were identified through synthesis of malaria elimination evidence describing residual transmission, malaria surveillance, prevention of re-establishment, community participation, and programme decision-making (3, 4, 9–11). The literature included points on potential communication and engagement functions (6, 7), while the social-listening and surveillance components were informed by studies applying social-network analysis and surveillance (12–14). The specific relationships presented in the table were then synthesized by the authors by mapping these documented information needs and social-media functions to the findings of the included studies.

**Table 1:** Malaria elimination challenges and potential information needs addressed by social media.

Malaria elimination challenge	Information gap	Potential social media application	Evidence question
Low preventive intervention use	Behavioral barriers	Social media communication	Can engagement influence behavior?
Residual transmission	Community perceptions of risk	Social listening	Can online signals complement surveillance?
Delayed care-seeking	Perceived barriers	Targeted messaging	Does social media communication improve timely care?
Community participation	Feedback and engagement	Two-way communication	Can social media engagement strengthen participation?
Vector-control concerns	Acceptance and concerns	Community monitoring	Can social listening inform implementation?
Vaccine introduction	Confidence and misinformation	Social media monitoring/communication	Can social media support vaccine implementation?
Misinformation	Emerging rumours	Social listening	Can misinformation be detected and addressed?
Digital inequality	Unequal reach	Audience analysis	Who is missed by digital interventions?

The central challenge, therefore, is to translate social media engagement into actionable public health intelligence, meaning actionable information obtained through social media engagement that provides insight relevant to malaria surveillance, community perceptions, information needs, misinformation, behaviors, or programme response (This is an author-derived operational definition informed by literature on digital epidemiology, public-health surveillance, and social listening (4, 12, 15), rather than a previously validated definition specific to malaria social media). In this context, social media functions not only as a communication channel but also as a source of information that can complement conventional public-health surveillance and inform programme responses. This requires connecting online information with validated surveillance data, interpreting signals within their epidemiological and social contexts, and establishing mechanisms to ensure findings influence malaria program decisions. This evidence-to-action pathway provides the basis for positioning social media as a complementary social media intelligence layer rather than a replacement for established malaria surveillance. The resulting information environment provides the conceptual context for examining whether and how social media can complement established malaria surveillance and implementation systems (Figure 1). This framework represents an author-derived synthesis rather than a formally validated theoretical model and integrates three evidence streams, i.e., established malaria surveillance and elimination principles (3–5, 9), public-health communication and engagement components (6, 7), and social-listening and intelligence components (15, 18–22). Evidence from intervention and programme studies included in this review further informed the links between knowledge and behavior, social-media engagement, programme response, and health outcomes (11–13, 30, 33–37). The final pathway and integration of these components were developed by the authors as a synthesis of these evidence streams.

**Figure 1:**
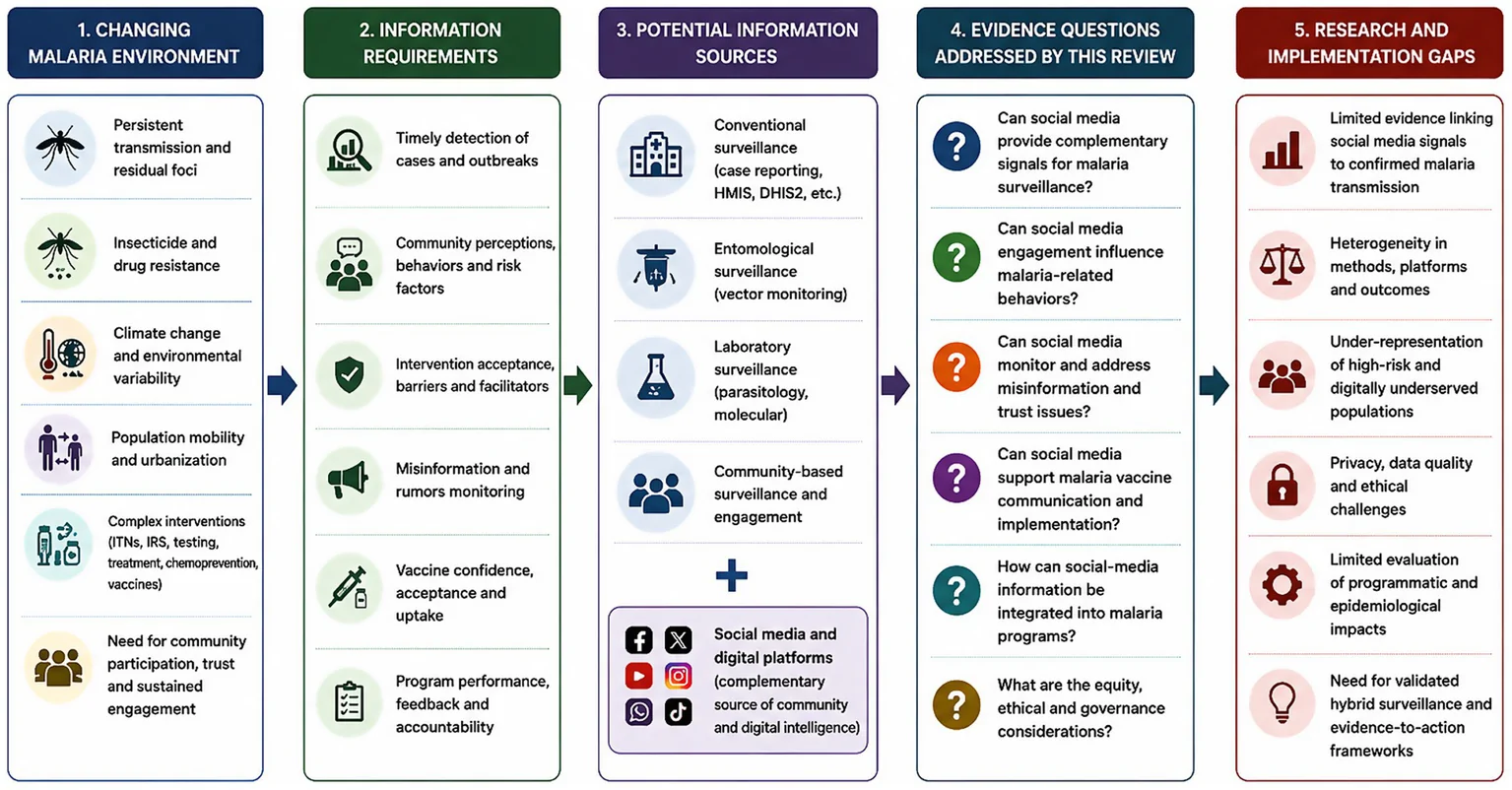
Conceptual context developed by the authors to illustrate the potential pathway from social-media engagement to public-health action in malaria elimination (ITNs Insecticide-Treated Nets; IRS Indoor Residual Spraying; HMIS Health Management Information System; DHIS2 District Health Information Software 2).

## 3 Methodology

### 3.1 Review rationale and design

This scoping review was conducted to systematically map and synthesize the available evidence on the use of social media in the elimination of malaria, identify opportunities, limitations, and gaps, and inform the integration of social media intelligence into malaria programs. A scoping review approach was selected because the objective was to characterize the distribution of existing evidence rather than estimate a single pooled intervention effect. The review was conducted in accordance with the methodological guidance for scoping reviews developed by the Joanna Briggs Institute (JBI) and was reported according to the Preferred Reporting Items for Systematic Reviews and Meta-Analyses extension for Scoping Reviews (PRISMA-ScR). The study protocol was registered with Open Science Framework (OSF) (DOI: https://doi.org/10.17605/OSF.IO/F4B6J).

### 3.2 Review framework

The review question and eligibility criteria were structured using the Population–Concept–Context (PCC) framework recommended for scoping reviews. The population component encompasses patients, caregivers, individuals, healthcare professionals, public health personnel, community health workers, malaria-control programs, and populations or communities living in or affected by malaria. The concept included the use of social media and digitally mediated social communication platforms, while the context comprised community, healthcare, public health, educational, governmental, research, and other relevant settings in malaria-endemic or malaria-affected regions, including national, regional, and cross-border malaria-control and elimination programs.

### 3.3 Definition of social media

For this scoping review, social media was defined as internet- or mobile-based platforms that enable users to create, share, or disseminate content, interact with others, and participate in social networks or online communities (16). This included Facebook, X/Twitter, Instagram, YouTube, TikTok, WhatsApp, Telegram, WeChat, LinkedIn, and Reddit. Stand-alone mobile health applications, SMS-only interventions, websites without social media functionality, email-based communication, telemedicine, and traditional mass media were not considered social media.

### 3.4 Eligibility criteria

Studies were eligible if they examined or reported the use, application, evaluation, implementation, or role of social media or social networking platforms in relation to malaria. Quantitative, qualitative, observational, mixed-methods, interventional, implementation, program-evaluation, and relevant descriptive studies were considered eligible. Studies focusing on populations or settings in malaria-endemic or malaria-affected regions were included. Studies were excluded when they were unrelated to malaria, did not involve social media or digitally mediated social communication, or provided insufficient information to establish a relevant malaria-related application. Editorials, commentaries, letters, conference abstracts without sufficient methodological or outcome information, and other non-evidence-based publications were also excluded.

### 3.5 Information sources

A comprehensive literature search was conducted in PubMed/MEDLINE, Web of Science Core Collection, Scopus, CINAHL, and Embase to provide broad coverage of public health, biomedical, social science, communication, and interdisciplinary literature relevant to malaria and social media. Additional records from reference-list screening included studies and relevant reviews, and targeted searches of relevant grey-literature sources and organizational websites, including those of international and national malaria-control agencies, were identified through Google Scholar.

### 3.6 Search strategy

A comprehensive search strategy was developed with controlled vocabulary terms, including MeSH and database-specific subject headings, and was combined with free-text keywords and synonyms. The searches covered the period from database inception to 31st July 2026 and were conducted without restricting studies by geographic region. Search terms for social media included *online communities, social networking (including individual platform names such as WhatsApp, LinkedIn, and Twitter/X), content-sharing platforms,* and *interactive messaging services*, while malaria-related terms included *malaria*, *malaria control*, *malaria vaccine, Plasmodium*, and *malaria elimination*. Boolean operators AND and OR were used to combine concepts, and database-specific syntax, proximity operators, truncation, and field searching were applied where appropriate.

A two-stage literature search strategy was implemented to ensure comprehensive coverage of both foundational and recently published studies relevant to the review objectives. To capture recently published evidence and maintain the accuracy of the review, a follow-up search was conducted using the same search terms to identify additional studies published between 1st February 2026 till 31st July 2026. This two-stage search approach was adopted to minimize the risk of omitting relevant studies and to ensure that the final review reflects the most up-to-date literature available at the time of analysis.

### 3.7 Study selection

All records retrieved from electronic databases and supplementary sources were imported into EndNote X9 citation organizer for reference management and duplicate removal. After deduplication, the remaining records were assessed for eligibility in a two-stage screening process. In the first stage, titles and abstracts were independently screened by XZ and GH against the predefined eligibility criteria. Records considered potentially relevant or for which eligibility could not be determined from the title and abstract were retained for full-text assessment. In the second stage, the full texts of potentially eligible records were independently assessed by the same authors. The reasons for exclusion at the full-text stage were recorded.

### 3.8 Screening and resolution of disagreements

The screening process was conducted independently by XZ and GH to minimize selection bias. Any disagreements were discussed between the two reviewers and resolved by consensus through reference to the predefined eligibility criteria and coding framework.

### 3.9 Data charting and quality assessment

The following information was charted from each included study: author and publication year; study design; geographic region; study population and sample size; malaria-related context; type of malaria activity addressed; social media platform or platforms used; purpose and mode of social-media use; intervention or communication characteristics; outcomes or indicators assessed; key findings; reported barriers and facilitators; implementation characteristics; and evidence gaps or limitations identified by the authors. Social media content, including tweets, posts, comments, hashtags, and other publicly available interactions, was considered a data source when systematically collected, sampled, coded, or computationally analyzed within an eligible study. Consistent with the purpose of a scoping review, formal critical appraisal of methodological quality was not undertaken.

### 3.10 Data extraction, synthesis and coding

Two reviewers independently screened the identified records and assessed potentially eligible full-text articles against the predefined eligibility criteria. Data from the included studies were extracted using a standardized coding framework developed for this review. The framework captured publication characteristics, publication year, geographical setting, study/evidence type, social-media platform, primary social-media role, malaria application, elimination relevance, and the highest demonstrated public-health action level. Social-media roles were categorized as health education/behavior change, health promotion, engagement, social listening, surveillance/intelligence, risk communication, misinformation monitoring, advocacy, community mobilisation, vaccine communication, or programme support. Public-health action was categorized according to the highest level demonstrated by each study: reach/engagement/knowledge, public-health intelligence, programme action/risk response, behavioral outcome, or health outcome. These categories were applied consistently across the included studies.

A descriptive content analysis was conducted to identify the scope, characteristics, and allocation of the publications included in the review. An inductive thematic approach was used to identify themes and synthesize the findings (17, 18). Screening and coding decisions were recorded using a structured coding framework and predefined exclusion reasons. Disagreements between the two reviewers were discussed and resolved by consensus through reference to the predefined eligibility criteria and coding framework. A formal statistical measure of intercoder reliability, such as Cohen’s kappa, was not calculated. The coding framework and operational definitions used for data extraction and synthesis are provided in Supplementary Material S1.

## 4 Results

### 4.1 Search results and study selection

A total of 214 records were identified through database and search engine searches. After removal of duplicates, 89 unique records remained for screening. Following title screening, 42 records were retained for further assessment. These records subsequently underwent full-text screening according to the predefined eligibility criteria, resulting in 30 studies being included in the final review. Eight reports were subsequently excluded because social media was only contextual or descriptive, its independent contribution could not be determined, the proposed application had not been evaluated, or malaria-specific evidence could not be extracted. Consequently, 30 unique studies were included, as presented in the PRISMA-ScR flow diagram (Figure 2).

**Figure 2:**
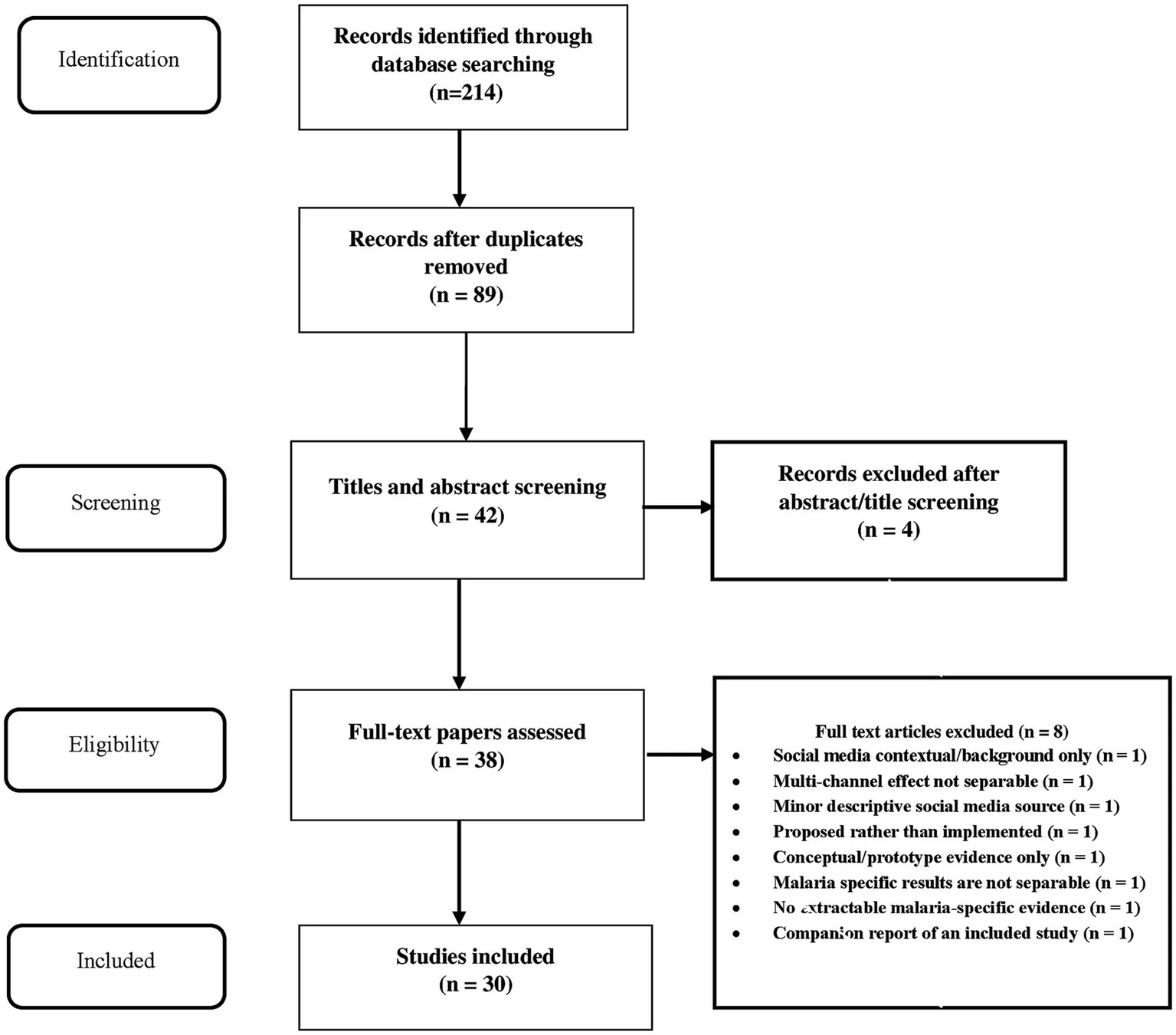
Preferred Reporting Items for Systematic Reviews and Meta-Analyses extension for Scoping Reviews (PRISMA-ScR) flow diagram.

### 4.2 Characteristics of the included evidence

A total of 30 studies met the eligibility criteria and were included in the final synthesis with their publication years ranges from January 2016 to July 2026. The evidence base has shown a marked increase in recent years, with 7 (23.3%) studies published in 2024 and 2025 each year, meaning that almost half of the included literature appeared in these 2 years alone. Geographically, more than half of the evidence (60%) involved African settings, including Niger, Ghana, Cameroon, Malawi, Kenya, Togo, Nigeria, Uganda, Senegal, and multi-country African initiatives. Eight (26.7%) were global or international in scope, three (10.0%) originated in China, and one (3.3%) evaluated a social media intervention in India. The evidence was methodologically heterogeneous, with 40.0% comprising intervention, campaign, program-evaluation, or response-oriented studies, and social listening, surveillance, network, or computational social-media analyses, respectively, as the largest category. Content and information-quality assessments accounted for four studies (13.3%), while qualitative implementation and observational communication-exposure studies accounted for one each (3.3%) (Table 2).

**Table 2:** Characteristics of the included studies.

Authors, year of publication, and country	Study design	Platform(s)	Malaria focus	Population/data source	Primary social media function	Main outcome	Action level
Li et al., 2016 (19), Niger	Randomized intervention	WeChat	Prevention/treatment	1,441 Chinese expatriates	Health education; behavior change	Improved malaria health literacy/KAP; health outcome evidence	Health outcome
Xue-Dong et al., 2018 (20), China	Pre/post campaign evaluation	WeChat	Imported malaria	Timber-industry overseas personnel	Health education; risk communication	Improved malaria knowledge; programme prevention evidence	Reach/knowledge
Wang et al., 2022 (21), China	Campaign evaluation	WeChat	Malaria awareness/prevention	13,169 campaign questionnaires	Health education; engagement	High participation and increased public-account reach	Reach/knowledge
Fung et al., 2017 (22), Global	Cross-sectional social-media analysis	Twitter/X	Malaria global-health discourse	Global-health tweets	Advocacy; discourse analysis	Mapped malaria topics and stakeholder priorities	Public-health intelligence
Lopreite et al., 2021 (12), Global	Social-network analysis	Twitter/X	Malaria organizations	Organizational accounts	Advocacy; networking	Mapped organizational connectivity and mobilisation potential	Public-health intelligence
Bornmann et al., 2020 (13), Global	Social-spatial analysis	Twitter/X	Malaria research attention	69,420 malaria-paper tweets with location data	Social-spatial sensing	Assessed geographic alignment of social-media attention and burden	Public-health intelligence
Ellis and Reichel, 2023 (15), Global	Text mining/topic modelling	Twitter/X	Malaria/Plasmodium discourse	Parasitology tweets	Social listening	Characterized topics, sentiment and hashtag trends	Public-health intelligence
Ong and Ahmad, 2024 (14), Global	Machine-learning social-media analysis	Twitter/X	Mosquito-borne diseases incl. malaria	6,263 cleaned tweets	Social listening; sentiment	Demonstrated disease-topic and sentiment analytics	Public-health intelligence
Tahir and Mehmood, 2024 (23), Global/Pakistan	Large-scale social-computing surveillance	Twitter/X	Malaria among epidemic-prone diseases	271 million tweets overall	Surveillance; misinformation intelligence	Identified temporal/spatial disease signals	Public-health intelligence
Cynthia et al., 2026 (24), Cameroon	NLP/sentiment analysis	Twitter/X; Facebook	RTS, S vaccine	Cameroonian social-media posts	Vaccine communication; social listening	42% negative, 40% positive, 18% neutral sentiment	Public-health intelligence
Grant et al., 2022 (25), Ghana	Qualitative implementation study	WhatsApp; social media	RTS, S vaccine	Health managers/workers	Misinformation; risk communication	Rumours contributed to caregiver concerns/refusals	Programme action
Walldorf et al., 2025 (26), Ghana, Kenya and Malawi	Programme evaluation	Social media	Vaccine implementation	Facilities, staff and caregivers	Communication; social mobilisation	Identified communication/misinformation issues in rollout	Programme action
TDR/WHO 2026 (8), Togo/regional Africa	Programme response report	WhatsApp; Facebook; TikTok	Malaria vaccine	Implementation programmes	Infodemic monitoring; crisis response	Detected and coordinated response to rapidly spreading rumours	Programme action
Adebisi et al., 2024 (27), Africa	Content-quality analysis	TikTok	Malaria vaccine	9 TikTok videos	Information dissemination; quality	Fair information quality despite engagement potential	Reach/knowledge
Omwando and Zhang 2021 (28), Africa	Mixed-methods content analysis	YouTube	Malaria information	Malaria-related YouTube content	Health information; content analysis	Mapped malaria-information themes	Reach/knowledge
Wang et al., 2025 (29), China	NLP/social-media analytics	Sina Weibo	Malaria discourse	81,010 Weibo posts	Social listening; engagement	Event-driven attention and differences by account type	Public-health intelligence
Linares-Rufo et al., 2021 (30), Global	Content analysis	Travel blogs/social networks	Travel malaria prevention	600 travel blogs	Information quality	Incomplete/inaccurate preventive advice identified	Reach/knowledge
Din et al., 2025 (31), Global	Content analysis	Travel blogs	Travel malaria prevention	100 blogs	Information quality	Inconsistent prevention and chemoprophylaxis advice	Reach/knowledge
Donati et al., 2024 (32), India	Cluster-randomized + individual experiments	Facebook; Instagram	Malaria prevention	8,257 + 1,542 participants	Behavior-change intervention	Effects on prevention/care seeking and malaria outcomes	Health outcome
Uganda LLIN campaign 2020, (33), Uganda	Mass campaign evaluation	Twitter/X; WhatsApp; Instagram; Facebook; YouTube	LLIN distribution/use	National campaign audiences	Social mobilization; communication	Influencer-driven reach and real-time campaign response	Programme action
Social and Behavior Change (SBC) Projects 2024, (34), Nigeria	Multi-year SBC programme	Facebook; Twitter/X; Instagram	Malaria prevention/fever case management within an integrated health programme	National digital audience	Health education/behavior-change communication and programme communication	Engagement and adaptive campaign management	Programme action/communication implementation
Catholic Relief Services (CRS) 2025, (35), Nigeria	Digital SBCC campaign	Facebook; Instagram	ITN use	Social-media audiences	Behavior-change communication	Brand-lift evidence for ITN importance/use	Behavioral outcome
US President’s Malaria Initiative (PMI) 2021, (36), African countries	Cross-country campaign	Facebook	Fever care seeking	Facebook users	Risk communication; care-seeking promotion	Reached >4.7 million users in first month	Reach/knowledge
Zero Malaria Starts with Me 2024, (37), Senegal/Africa	Advocacy campaign retrospective	Facebook/social media	Elimination advocacy	Public/stakeholder audiences	Advocacy; mobilisation	Amplified elimination advocacy and stakeholder mobilisation	Reach/knowledge
Chitsime 2025, (38), Malawi	Mixed-methods campaign evaluation	Facebook	Prevention/control behaviors	122 posts; comments/reactions	Celebrity-led health promotion	Substantial engagement; response varied by framing/credibility	Reach/knowledge
Mwebesa et al., 2025 (39), Uganda	Observational communication analysis	Social media within multiple channels	ITN use	8,868 women; 6,915 children	Health communication exposure	Message exposure associated with ITN use; social-media effect not separable	Behavioral outcome
Africa Infodemic Response Alliance, 2023, (40), Ghana, Nigeria, South Africa and South Sudan	Infodemic surveillance report	Facebook/social media	R21 vaccine misinformation	Online posts/comments	Infodemic monitoring; risk communication	Tracked misinformation narratives and engagement after vaccine clearance	Public-health intelligence
Africa Infodemic Response Alliance, 2024, (41), Africa	Infodemic surveillance report	Social media/influencer networks	RTS, S misinformation	Online vaccine narratives	Infodemic monitoring; prebunking	Identified misinformation risks and recommended proactive response strategies	Public-health intelligence
Africa Infodemic Response Alliance, 2024, (42), Nigeria	Infodemic surveillance report	Social media; Twitter/X	R21 rollout misinformation	Online vaccine posts	Infodemic monitoring; social listening	Documented false mRNA claims and distrust narratives	Public-health intelligence
Africa Infodemic Response Alliance, 2025, (43), Togo, Nigeria, Ghana and Kenya	Infodemic surveillance report	Facebook; Twitter/X; TikTok	RTS, S/R21 vaccine debate	Online vaccine narratives	Infodemic monitoring; cross-country listening	Tracked persistent cross-border malaria-vaccine misinformation	Public-health intelligence

### 4.3 Social media platforms used in malaria-related activities

A broad range of social media platforms was represented, reflecting substantial diversity in social media approaches to malaria communication and surveillance. Twitter/X was the most frequently represented platform (36.7%), followed by Facebook (33.3%). Instagram was represented in four evidence units (13.3%), while WeChat, WhatsApp, and TikTok each appeared in three (10.0%). YouTube and blogs were each represented in two studies (6.7%), and Sina Weibo in one study (3.3%). Several programs adopted multiplatform approaches, particularly in large-scale campaigns and malaria vaccine communication initiatives. Consequently, platform frequencies were not mutually exclusive.

### 4.4 Health education, awareness, and behavior-change communication

The included studies demonstrated several distinct but overlapping functions of social media across the malaria-elimination continuum. Health education and behavior-change communication represented the most common primary application of social media, accounting for 10/30 (33.3%) studies. A randomized intervention among Chinese expatriates in Niger demonstrated improvements in malaria-related knowledge, attitudes, practices, and health literacy following the delivery of malaria information through WeChat (19). A subsequent Chinese study targeting personnel working overseas similarly reported improved malaria knowledge following WeChat publicity, while a National Malaria Day WeChat campaign attracted more than 13,000 questionnaire responses and substantially increased the participating public account’s followers (21).

Large-scale social-media campaigns extended this approach to malaria-endemic populations. The Facebook/Instagram intervention in India evaluated digitally delivered malaria-prevention messages through field experiments involving more than 8,000 participants and examined bed-net use, treatment-seeking, and malaria-related outcomes (32). Large-scale African programs similarly used Facebook, Instagram, Twitter/X, and other channels to promote insecticide-treated net use, fever-related care-seeking, and malaria-prevention behaviors (40–43).

### 4.5 Misinformation, vaccine communication, and program response

Qualitative evidence from Ghana and the multi-country (Ghana, Kenya, and Malawi) documented the misinformation disseminated through social media channels containing rumours concerning vaccine safety, infertility, death, and experimentation, which contributed to caregiver concerns and vaccine refusals (26). Sentiment analysis of RTS, S-related posts from Cameroon revealed a divided online response: 42% negative, 40% positive, and 18% neutral, illustrating the potential value of platform-based monitoring for assessing public confidence during the vaccine rollout (24). Reports from 2023 to 2025 documented misinformation surrounding R21 and RTS, S across Ghana, Nigeria, South Africa, South Sudan, Togo, Kenya, and other African settings, including distrust of vaccine development, concerns about experimentation, false claims about vaccine technologies, and the cross-border circulation of misleading content (25, 27, 44,).

### 4.6 Social media engagement to public health intelligence

To examine whether social media activities extended beyond communication metrics, the included studies were classified by their highest demonstrated level of public health action. Twelve studies (40.0%) generated public-health intelligence, including social listening, surveillance, sentiment analysis, network analysis, spatial monitoring, and infodemic intelligence. Ten studies (33.3%) primarily addressed reach, engagement, or knowledge outcomes. Four studies (13.3%) demonstrated translation into program action or risk response. Only two studies (6.7%) demonstrated behavioral outcomes, and two (6.7%) reached malaria-related health outcomes (Figure 3). Twitter/X was particularly prominent in this domain. Studies analyzed malaria-related hashtags, organizational networks, geographic patterns of malaria research attention, public sentiment, and disease-related conversations. Analysis of 81,010 malaria-related Sina Weibo posts showed that public attention was strongly event-driven and differed between official and non-official accounts (29). Program evaluations in Uganda, Ghana, and other African settings also demonstrated how social-media intelligence and communication could inform implementation and response activities.

**Figure 3:**
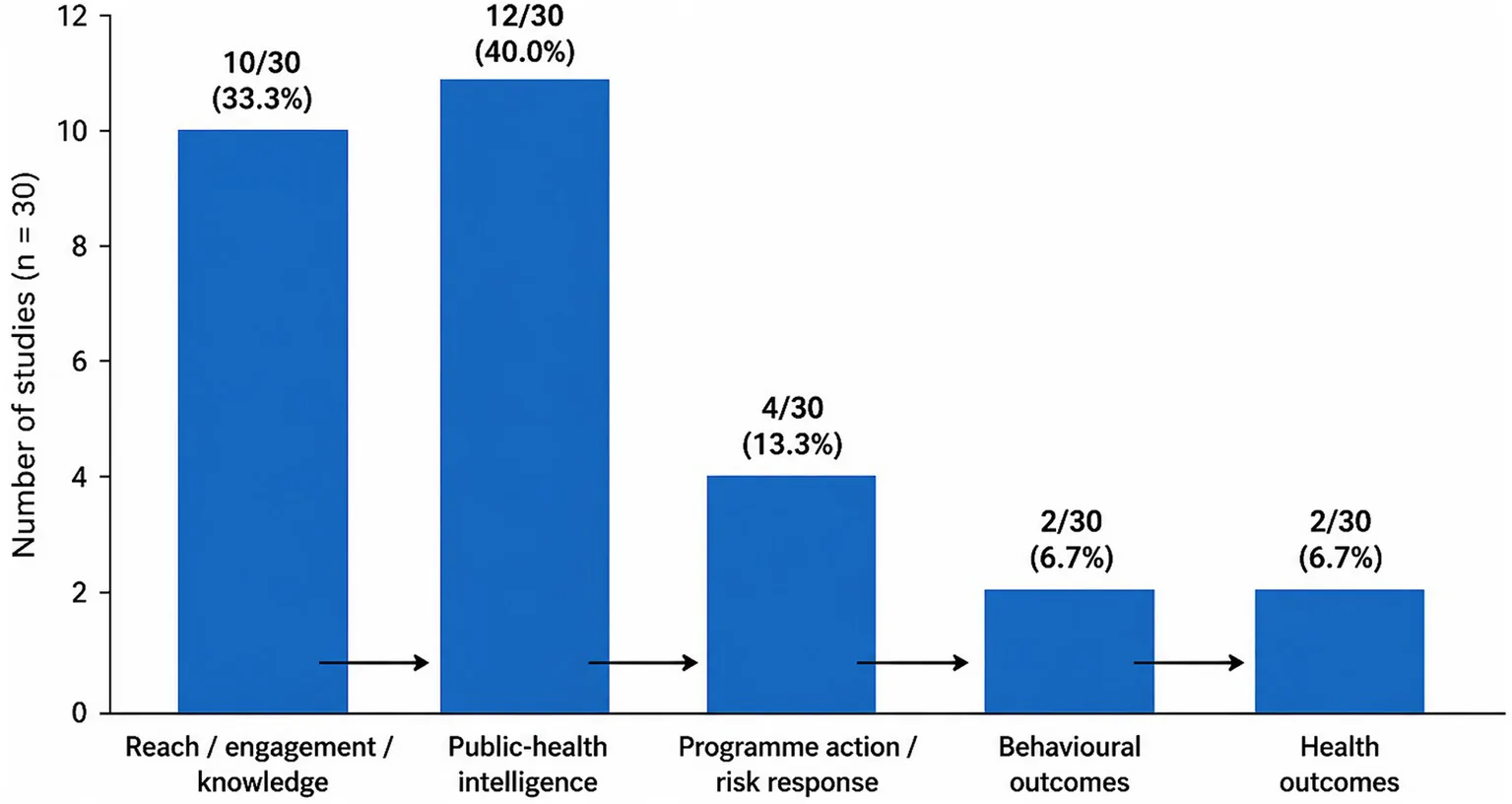
From social media engagement to public health action in malaria elimination.

## 5 Discussion

### 5.1 Summary of findings

This scoping review provides a comprehensive synthesis of the role of social media across malaria control and elimination and, importantly, examines the extent to which social media engagement is translated into public-health action. Through the studies included, social media served multiple and increasingly sophisticated functions, which varied and emphasized that surveillance, community engagement, and context-specific responses are integral to malaria elimination and prevention of re-establishment. Online conversations can reveal public concerns, emerging misinformation, geographic patterns of attention, communication gaps, and shifting attitudes toward malaria interventions. At the same time, the findings reveal an important engagement-to-action gap, demonstrating that the current evidence is substantially stronger for what social media can communicate, detect, and characterize than for what it can independently change in terms of malaria prevention behaviors, morbidity, or transmission. This distinction is important because views, likes, shares, comments, and even improvements in knowledge should not automatically be interpreted as evidence of public-health impact.

Taken together, the findings suggest that the contribution of social media to sustainable malaria elimination is best conceptualized as a broad range rather than a single intervention, starting with engagement and communication, leading to the generation of public health intelligence, proceeding to behavioral change, with the result as health impact. The evidence base is currently concentrated in the first three stages of this pathway. This conceptual distinction represents an important contribution to the present review because it prevents reach from being equated with elimination impact and identifies the critical evidence gap that future implementation research must address.

### 5.2 Comparison with existing literature

Health education and behavior-change communication represented one of the most established applications of social media in the included literature. These findings are consistent with the broader role of social and behavior change communication (SBCC) within malaria programs (7). WHO-supported malaria strategies recognize that communication, community participation, and advocacy are important for maintaining appropriate prevention, diagnosis, treatment, and surveillance behaviors, including in settings seeking to prevent re-establishment of transmission (6). WHO defines malaria surveillance as the systematic collection, analysis, and interpretation of malaria-related information for planning, implementation, and evaluation, and stresses that strong surveillance systems are fundamental to identifying affected populations and tailoring interventions (45). Social media intelligence should therefore be viewed as a complementary layer of information, rather than a replacement for epidemiological, entomological, or case-based surveillance.

Social media potentially expands conventional SBCC in several ways. Social media platforms permit rapid dissemination of information to large audiences, repeated exposure to messages, audience segmentation, interactive communication, and adaptation of campaign content in response to engagement data. They also provide access to populations that are difficult to reach through facility-based communication alone, including mobile workers, travelers, and geographically dispersed populations. Literature on malaria behavior-change communication similarly argued that communication becomes increasingly important as countries progress toward elimination because programs must reach changing risk groups, communicate evolving diagnostic and treatment recommendations, and maintain preventive behaviors as perceived malaria risk declines (3, 9). Nevertheless, increased exposure to information does not guarantee behavioral change. This is because individuals may understand recommended malaria-prevention practices but fail to adopt them consistently because behavior is also shaped by trust, physical discomfort, social norms, health-system accessibility, and contextual constraints.

The value of social media surveillance, therefore, lies less in predicting malaria cases directly than in adding a social and behavioral layer to malaria intelligence systems. WHO is already expanding digital approaches to malaria surveillance through tools such as DHIS2 malaria modules and national malaria data repositories, emphasizing standardized data collection, visualization, and use for decision-making (11). Such triangulation may provide programs with a more comprehensive picture of not only where malaria transmission is occurring, but also how communities are perceiving and responding to elimination interventions. In this case, important methodological challenges remain. Social-media users are not representative of entire populations, and platform use varies substantially by age, socioeconomic status, geography, connectivity, and national context. Public posts also represent only a fraction of online communication; important malaria-related narratives may circulate through private or encrypted channels that researchers cannot systematically observe. Furthermore, algorithms governing content visibility can influence which posts receive attention, while automated accounts, coordinated campaigns, and repeated posting may distort apparent levels of public concern. Consequently, ethical considerations, limited standardization and validation of malaria-specific indicators (engagement metrics, sentiment, misinformation prevalence, geographic and temporal patterns, and associations with malaria-related outcomes) for assessing the surveillance and public-health utility of social-media data are additional challenges (10, 11).

The introduction and scale-up of malaria vaccines have created a new communication environment in which social media can simultaneously support vaccine uptake and amplify misinformation. The prominence of this theme in recent studies indicates that digital information environments are becoming increasingly relevant to malaria vaccine implementation (44). Included studies and surveillance reports documented concerns relating to vaccine safety, effectiveness, distrust of institutions, experimentation narratives, and misleading claims about vaccine technologies. WHO recognizes that infodemics can generate confusion, promote risky behavior, and undermine trust in health authorities, while digital connectivity enables both accurate and misleading information to spread rapidly (43), creating an important distinction between misinformation monitoring and infodemic management.

This represents the central argument of the present review that social media engagement becomes valuable to malaria elimination when it generates intelligence that changes public-health action. The prominence of public-health intelligence nevertheless represents an important development. Counting vaccine-related posts or measuring sentiment provides descriptive information, whereas identifying a rapidly spreading harmful narrative provides intelligence that, in response, represents public-health action (6, 14, 33). In this sense, the pathway should not necessarily be interpreted as a simple linear hierarchy in which intelligence is inferior to behavioral outcomes. Intelligence may itself constitute an important program resource when it informs decisions that would otherwise be made with limited information about community perceptions. Establishing whether those actions subsequently improve vaccine confidence or uptake constitutes the next stage of evidence.

### 5.3 Methodological gaps and priorities for future research

Despite increasing interest in social media, the evidence base remains methodologically uneven. A major limitation is the continued reliance on social media engagement metrics as endpoints, as they provide little information about whether users understood a message, changed their behavior, or accessed malaria services. Future intervention studies should therefore establish *a priori* outcome framework that distinguishes between program actions and health outcomes.

A second priority is that social-listening methodologies require greater standardization and transparency. Studies should report platforms, search terms, languages, data-collection periods, inclusion and exclusion rules, procedures for identifying automated or duplicate content, sentiment-classification methods, validation procedures, and ethical safeguards. Reproducible reporting will become increasingly important as machine learning and artificial intelligence are used to analyze large volumes of malaria-related digital content.

Maintaining attention during periods of declining malaria transmission is another important challenge for sustainable elimination. As malaria incidence decreases, communities may perceive malaria as a less immediate threat, potentially reducing attention to preventive behaviors and demand for malaria-related information, creating a potential communication paradox. Social media and other communication channels could help maintain awareness between periods of high transmission by providing continuous, locally relevant information and reinforcing preventive behaviors. However, communication activities should remain proportionate to epidemiological risk and should be coordinated with routine malaria surveillance rather than relying on online engagement alone.

Fourth is a stronger causal evaluation. Many observational studies cannot determine whether social media exposure independently produces an observed outcome. Randomized digital interventions, cluster-randomized designs, controlled before-and-after studies, interrupted time-series analyses, and natural experiments should be considered where feasible. Hybrid effectiveness–implementation designs may be particularly valuable because they can assess both whether a digital intervention works and how it can be integrated into routine malaria programs.

Fifth, greater attention is required to equity and representativeness. Online populations may systematically differ from those experiencing the greatest malaria burden. Future studies should examine those voices that are represented in social media datasets and those that are absent, particularly in populations with limited connectivity, displaced populations, and groups with lower digital literacy. Digital strategies that systematically overlook these populations could inadvertently widen communication inequalities.

Although this review focused only on social media platforms, mobile communication technologies such as SMS also represent an important channel for rapid dissemination of malaria-related information. SMS-based communication can be particularly relevant in settings where broadband connectivity, smartphone ownership, or access to social-media platforms is limited. However, SMS was outside the predefined scope of this review because it does not provide the social-networking and interactive characteristics used to define social media in our eligibility criteria. Future research should examine how social media, SMS, mobile applications, community radio, and interpersonal communication can be integrated into complementary digital and non-digital communication strategies for malaria elimination.

Finally, future studies should determine whether social media interventions are cost-effective and sustainable on a program scale. Very little current evidence establishes the resources required to maintain social listening, produce locally adapted content, moderate online engagement, and translate intelligence into program response. Economic evaluations and implementation-cost studies would therefore strengthen the case for integrating digital approaches into national malaria programs.

### 5.4 Strengths and limitations

This scoping review has several strengths. First, it examines social media across the broader malaria elimination, instead of restricting the synthesis to health education or vaccine communication. The included studies, by integrating evidence, identified the changes in the role of social media over time, particularly in disseminating information toward generating public-health intelligence and supporting program response. A further strength is the analytical distinction between social media engagement and public health action, highlighting the substantial gap between digital activity and demonstrated population-health effects, and providing a framework that may be useful for evaluating future malaria interventions. Moreover, the review incorporates both peer-reviewed research and relevant program and institutional evidence that was particularly important for emerging areas like infodemic surveillance, where operational experiences may be documented by public-health organizations before appearing in conventional academic literature. Restricting the review exclusively to peer-reviewed studies could therefore have omitted recent developments in social listening and real-time misinformation response.

Several limitations should nevertheless be considered. First, substantial heterogeneity existed in the included studies, which is expected as an emerging interdisciplinary field, but precluded quantitative pooling and limited direct comparison of effect sizes across studies. Second, much of the literature relied on observational designs, program reports, platform analytics, or cross-sectional assessments, limiting causal inference regarding whether social media exposure itself produced improvements in malaria prevention or control. Even where behavioral or health changes were reported, the independent contribution of social media may be difficult to distinguish from concurrent activities. Platform and population selection biases are unavoidable considerations. Social media users do not necessarily represent the population most affected by malaria, but rather communities with limited internet connectivity, smartphone access, or digital literacy. Publicly accessible social media data may further overrepresent highly active users while underrepresenting individuals communicating through private or encrypted channels. The review’s focus on social media platforms also means that other important communication modalities, including SMS, mobile applications, radio and interpersonal communication, were not comprehensively mapped. Consequently, the findings should not be interpreted as representing the entire digital or communication ecosystem for malaria elimination.

Changes in social media platforms may reduce the comparability of studies conducted at different time points. The transition from Twitter to X and the increasing prominence of TikTok and encrypted messaging platforms illustrate the dynamic nature of social media engagement. Consequently, some platform-specific findings may have limited long-term generalizability. Finally, despite the broad search approach and updated search conducted before final synthesis, relevant evidence may have been missed because of inconsistent terminology across digital-health, communication, and malaria literature, restrictions in database indexing, rapidly emerging grey literature, and the multilingual nature of social media research.

## 6 Conclusion

Social media is emerging as a multifaceted component of malaria control and elimination, with the scoping review identifying a substantial engagement-to-action gap, demonstrating communication, engagement, or intelligence-generation outcomes, but direct evidence demonstrating translation into behavioral change or measurable malaria health outcomes remains comparatively limited. It is not considered a stand-alone intervention or a substitute for established surveillance and health-system infrastructure, but rather functions as a complementary bridge between communities, information systems, and public-health response. Integrating structured social listening with epidemiological surveillance, community engagement, behavior-change programs, and malaria-vaccine implementation may allow programs to identify emerging concerns, respond to misinformation, and adapt interventions more rapidly to local needs.

## Data Availability

The original contributions presented in the study are included in the article/Supplementary material, further inquiries can be directed to the corresponding author.

## References

[1] Aftab M Qureshi AW Aftab Z Babar A. Investigating mosquito borne parasitic diseases and vector control strategies. Res. Consort. Arch. (2026) 4:2948–60. doi: 10.5281/zenodo.20806629

[2] Yin J Zhang T Liu Y Li J Huang Y. Prevention of re-establishment of malaria transmission in China: insights from the world malaria report 2025. Zoonoses (Basel). (2025) 6:986. doi: 10.15212/zoonoses-2026-0011

[3] Elendu C Amaechi DC Elendu RC Saggi SK Elendu TC Amaechi EC . Comparative analysis of Egypt’s malaria elimination strategies and implementation science: pathways to achieve malaria-free status for other African countries. PLoS One. (2025) 20:e0329550. doi: 10.1371/journal.pone.0329550, 40743245 PMC12312880

[4] Organization WH. Malaria Surveillance, Monitoring and Evaluation: a Reference Manual. 2nd Edn. Geneva: World Health Organization (2025).

[5] Abdul Rahim FA Mahmud MAF Abdul Mutalip MH Yoep N Aminuddin MAH Mohd Ngesom AM. A scoping review of community knowledge in malaria prevention and control programmes. PLoS One. (2025) 20:e0328703. doi: 10.1371/journal.pone.0328703, 40690438 PMC12279157

[6] Thapliyal K Thapliyal M Thapliyal D. "Social media and health communication: a review of advantages, challenges, and best practices". In: Emerging Technologies for Health Literacy and Medical Practice (2024). p. 364–84.

[7] Begba UW Abah MA Oladosu MA Agida OD Oduah IV Rimam AV . The impact of social media on public health: a review of the evidence on health promotion, disease prevention, and health communication. J. e-Sci. Lett. (2026) 7:55–62. doi: 10.51470/esl.2026.7.1.55

[8] TDR F research on diseases of poverty. Tackling social media Influencers Spreading Rumours on the malaria Vaccines. (2026). Available online at: https://tdr.who.int/newsroom/news/item/23-04-2026-countering-social-media-influencers-spreading-rumours-on-the-malaria-vaccines (Accessed August 31, 2026).

[9] Hemingway J Shretta R Wells TNC Bell D Djimdé AA Achee N . Tools and strategies for malaria control and elimination: what do we need to achieve a grand convergence in malaria? PLoS Biol. (2016) 14:e1002380. doi: 10.1371/journal.pbio.1002380, 26934361 PMC4774904

[10] Okereke PU Okereke OW Ogunyinka BO Aluele SI Tiamiyu RA Afolabi MA . Mobile app-based malaria surveillance and vector control integration: a scoping review of evidence from endemic settings. Malar J. (2025) 24:412. doi: 10.1186/s12936-025-05656-4, 41257723 PMC12628951

[11] Stratil AS Canana N Rodrigues M Armando S Gomane S Zandamela E . Improving the quality and use of malaria surveillance data: results from evaluating an integrated malaria information storage system at the health facility level in selected districts in Mozambique. Am J Trop Med Hyg. (2024) 112:59–65. doi: 10.4269/ajtmh.23-0138, 38266287 PMC11720678

[12] Lopreite M Puliga M Riccaboni M De Rosis S. A social network analysis of the organizations focusing on tuberculosis, malaria and pneumonia. Soc Sci Med. (2021) 278:113940. doi: 10.1016/j.socscimed.2021.113940, 33940437

[13] Bornmann L Haunschild R Patel VM. Are papers addressing certain diseases perceived where these diseases are prevalent? The proposal to use twitter data as social-spatial sensors. PLoS One. (2020) 15:e0242550. doi: 10.1371/journal.pone.0242550, 33216816 PMC7678968

[14] Ong SQ Ahmad H. Tracking mosquito-borne diseases via social media: a machine learning approach to topic modelling and sentiment analysis. PeerJ. (2024) 12:e17045. doi: 10.7717/peerj.17045, 39670104 PMC11636683

[15] Ellis JT Reichel MP. Twitter trends in# parasitology determined by text mining and topic modelling. Curr Res Parasitol Vector-Borne Dis. (2023) 4:100138. doi: 10.1016/j.crpvbd.2023.100138, 37670843 PMC10475476

[16] El Ouirdi M El Ouirdi A Segers J Henderickx E. Social media conceptualization and taxonomy: a Lasswellian framework. J Creat Commun. (2014) 9:107–26. doi: 10.1177/0973258614528608

[17] Ahmed SK Mohammed RA Nashwan AJ Ibrahim RH Abdalla AQ Ameen BMM . Using thematic analysis in qualitative research. J Med Surg Public Health. (2025) 6:100198. doi: 10.1016/j.glmedi.2025.100198

[18] Naeem M Ozuem W Howell K Ranfagni S. A step-by-step process of thematic analysis to develop a conceptual model in qualitative research. Int J Qual Methods. (2023) 22:16094069231205788. doi: 10.1177/16094069231205789, 34811878

[19] Li W Han LQ Guo YJ Sun J. Using WeChat official accounts to improve malaria health literacy among Chinese expatriates in Niger: an intervention study. Malar J. (2016) 15:567. doi: 10.1186/s12936-016-1621-y, 27881122 PMC5121985

[20] Xue-Dong W Ling J Feng H Bin L Yi R. Role of WeChat publicity in control of imported malaria from abroad in timber industry. Zhongguo Xue Xi Chong Bing Fang Zhi Za Zhi. (2018) 30:688–90. doi: 10.16250/j.32.1374.2018202, 30891987

[21] Wang Y Li C Zhang J Yang M Zhu G Liu Y . Using social media for health education and promotion: a pilot of WeChat-based prize quizzes on China national malaria day. Malar J. (2022) 21:381. doi: 10.1186/s12936-022-04404-2, 36514171 PMC9745723

[22] Fung ICH Jackson AM Ahweyevu JO Grizzle JH Yin J Tse ZTH . #Globalhealth twitter conversations on#malaria,#HIV,#TB,#NCDS, and#NTDS: a cross-sectional analysis. Ann Glob Health. (2017) 83:682–90. doi: 10.1016/j.aogh.2017.09.006, 29221545

[23] Tahir B Mehmood MA. Leveraging social computing for epidemic surveillance: a case study. Big Data Res. (2024) 38:100483. doi: 10.1016/j.bdr.2024.100483

[24] Cynthia AN Njuaka M Koomson N Fogap NZ. Assessing RTS, S malaria vaccine rollout perception in Cameroon: sentiment analysis from X and facebook using hugging face. Health Informatics J. (2026) 32:14604582261416864. doi: 10.1177/14604582261416864, 41506533

[25] Grant J Gyan T Agbokey F Webster J Greenwood B Asante KP. Challenges and lessons learned during the planning and early implementation of the RTS, S/AS01E malaria vaccine in three regions of Ghana: a qualitative study. Malar J. (2022) 21:147. doi: 10.1186/s12936-022-04168-9, 35550113 PMC9096766

[26] Walldorf JA Mayers GF Amponsa-Achiano K Jalang’o R Chisema M Khalayi L . Post introduction evaluation (PIE) of the malaria vaccine introduced in three pilot countries (Ghana, Kenya, and Malawi) in 2021. Malar J. (2025) 24:333. 10.1186/s12936-025-05590-5 PMC12522374 41088219

[27] Adebisi J Adesina M Oladele R Akinwola M. Malaria Vaccine Information in Africa: an Analysis of the Quality and Engagement of Tiktok Videos. Geneva: Geneva Health Forum 2024 (2024). Available online at: https://ghf.unige.ch/event/1/contributions/88/ (Accessed September 1, 2026).

[28] Omwando B Zhang J. Analysis of Malaria Information on a Social Media P Latform International Conference on Human-Computer Interaction.Springer (2021). p. 298–316.

[29] Wang N Zhao X Li C Mao Y. Event-driven dynamics, regional disparities, and netizen engagement: characterizing and exploring malaria discourse on Sina Weibo for enhanced health communication. Malar J. (2025) 24:429. doi: 10.1186/s12936-025-05664-4, 41299457 PMC12659473

[30] Linares-Rufo M Santos-Larrégola L Hernández-de-Mora MG Ramos-Rincón JM. Contents and quality of travel tips on malaria in English and Spanish travel blogs. Malar J. (2021) 20:342. doi: 10.1186/s12936-021-03864-2, 34399776 PMC8365983

[31] Din S Bishay R Asad U Flaherty GT. Content analysis of malaria-related health information published in online travel blogs. Travel Med Infect Dis. (2025) 67:102879. doi: 10.1016/j.tmaid.2025.102879, 40749952

[32] Donati D Rao N Orozco-Olvera VH Boudet AMM. Can Facebook ads Prevent Malaria? Two Field Experiments in India.World Bank (2024).

[33] Alliance for Malaria Prevention. Using social Media to Improve Campaign Efficiency: The Ugandan Experience. (2020). Available online at: https://allianceformalariaprevention.com/using-social-media-to-improve-campaign-efficiency-the-ugandan-experience/ (Accessed September 1, 2026).

[34] Johns Hopkins Center for Communication Programs. Nigeria Mass Media and Digital Interventions. (2024). Available online at: https://thecompassforsbc.org/sbcc-tools/nigeria-mass-media-and-digital-interventions (Accessed September 1, 2026).

[35] Nweke F. Digitalizing Malaria Mass Distribution Campaigns Handbook 2.0. (2025).

[36] Panjabi R. US President’s Malaria Initiative (PMI). (2021).

[37] Wallen J. From Grassroots to Global: Lessons from the Zero Malaria Starts with Me Campaign 2014–2024. (2024) Available online at: https://www.speakupafrica.org/from-grassroots-to-global-lessons-from-the-zero-malaria-starts-with-me-campaign-2014-2024/ (Accessed September 1, 2026).

[38] Chitsime A. Using Celebrity Social Media Campaigns to Promote Covid-19 Vaccine Uptake and Malaria Prevention and Control Behaviours in Malawi.1st National Conference on Health Promotion - Malawi (2025).

[39] Mwebesa E Agbi DM Iha DT Agasa LO Mwangi A. Effect of exposure to malaria messages on insecticide-treated net use among women and under-five children in Uganda: a propensity score matched analysis. Malar J. (2025) 24:386. doi: 10.1186/s12936-025-05629-7, 41214664 PMC12604199

[40] Karam EM Ho E Africa Infodemic Response Alliance (2023). Available online at: https://www.afro.who.int/sites/default/files/2023-04/AIRA%20Infodemic%20Trends%20Report%2014-20%20April%202023.pdf (Accessed September 1, 2026).

[41] Karam EM Ho E Africa Infodemic Response Alliance. (2024).

[42] El Moussaoui Y Inigo RL Africa Infodemic Response Alliance. (2024). Available online at: https://www.afro.who.int/sites/default/files/2024-12/AIRA%20Infodemic%20Trends%20Report%2019%20November-3%20December%202024.pdf (Accessed September 1, 2026).

[43] WHO. Africa Infodemic Response Alliance. (2025). Available online at: https://www.afro.who.int/sites/default/files/2025-09/Infodemic%20Insights%20report%201-15%20september%2C%202025-%20170.pdf (Accessed September 1, 2026).

[44] Adjei MR Tweneboah PO Bonsu G Baafi JV Amponsa-Achiano K Asiedu-Bekoe F . An assessment of Ghana’s pilot of the RTS, S malaria vaccine implementation programme; 2019–2021: a retrospective study. Malar J. (2024) 23:290. doi: 10.1186/s12936-024-05113-8, 39334244 PMC11438052

[45] Organization WH. Global Framework for the Response to malaria in urban Areas.World Health Organization (2022).

