# Supplementary material for: From social media engagement to public health action: the role of social media in sustainable malaria elimination
Source: Front Public Health. 2026 Oct 30;14:1987769. doi: 10.3389/fpubh.2026.1987769 (PMC13759760; doi:10.3389/fpubh.2026.1987769)
Supplement: Supplementary file 1 [file Supplementary_file_1.docx]

**Supplementary File**

**Coding Framework and Operational Definitions**

# Table S1. Variables, codes, and operational definitions

| **Variable** | **Code / recorded field** | **Operational definition** |
| --- | --- | --- |
| Study identification | Study ID | Unique identifier assigned to each included study (e.g., S01, S02, S03). |
| Bibliographic information | Author; publication year; title; source | Bibliographic information recorded directly from the included publication/report. |
| Country / setting | Country / setting | Country or geographic setting represented by the included evidence; multi-country/global studies were recorded as such. |
| Evidence type | Evidence / study type | The study or evidence design/type reported by the source, using the terminology applicable to that record. |
| Source type | Source type | Publication or evidence source category as recorded in the master sheet (e.g., peer-reviewed article, programme/project report, case study/report). |
| Social-media platform | Platform | Platform(s) explicitly examined, analysed, or used in the study; multiple platforms were retained when applicable. |
| Primary social-media role | Primary role | Main role of social media addressed in the study, based on the reported purpose or application rather than assumed potential. |
| Malaria application | Malaria application / topic | Specific malaria-related activity, behaviour, programme component, communication issue, surveillance function, or other application examined by the study. |
| Public health intelligence | Public health intelligence | Actionable information obtained through social-media engagement that provides insight relevant to malaria surveillance, community perceptions, misinformation, information needs, behaviours, or programme response |
| Engagement / communication outcome | Engagement outcome | Reported form or measure of audience engagement, communication, interaction, reach, participation, or response, where available. |
| Surveillance / social listening | Surveillance function | Whether and how social-media information was used for malaria surveillance, social listening, monitoring, signal identification, or related evidence generation. |
| Evidence-to-action linkage | Action linkage | Highest level of public-health action explicitly demonstrated or linked to social-media evidence in the source. This was not inferred when the source only discussed potential use. |
| Public-health action | Demonstrated action | Concrete action, decision, programme response, communication response, or implementation activity reported in association with the social-media evidence. |
| Misinformation / trust | Misinformation / trust | Whether misinformation, rumours, trust, credibility, or related information disorders were examined or addressed. |
| Behaviour-change component | Behaviour change | Whether the study addressed a malaria-related behaviour or behaviour-change communication component. |
| Community engagement | Community engagement | Whether social media was used to engage, mobilize, communicate with, or obtain feedback from communities or target audiences. |
| Malaria-vaccine component | Vaccine implementation | Whether the evidence addressed malaria-vaccine communication, confidence, acceptance, demand, implementation, or related public-health activity. |
| Conventional-surveillance integration | Data integration | Whether social-media information was explicitly combined, compared, triangulated, or linked with conventional epidemiological, surveillance, diagnostic, entomological, geospatial, or other public-health data. |
| Key finding | Key finding | Concise extraction of the principal malaria-relevant finding reported by the included source. |
| Inclusion decision | Inclusion | Final decision after screening and eligibility assessment; all records in this supplementary coding file represent included studies. |
| Exclusion reason | Exclusion reason | For the screening database, the documented reason for exclusion when applicable; excluded records were not included in the final synthesis. |

# Coding procedures

- Two reviewers independently screened, extracted, and coded the included evidence. Disagreements were resolved through discussion and consensus; no formal intercoder reliability statistic was calculated.
- Coding was based on information explicitly reported in each included source. The framework did not treat theoretical or proposed uses of social media as demonstrated outcomes.
- Where a study covered multiple platforms, countries, applications, or functions, the relevant information was retained in the extraction fields. The primary category reflected the principal focus of the evidence.
- For variables not reported by a source, the corresponding field was recorded as not reported/unclear rather than assuming absence.
- The public-health action field was coded according to the highest level of action explicitly demonstrated or linked in the source. This distinction was used to avoid overstating the contribution of social media to malaria elimination.
- The coded variables supported the descriptive and narrative synthesis of surveillance/social listening, communication and engagement, behaviour change, misinformation/trust, malaria-vaccine implementation, data integration, computational/AI methods, and translation of social-media evidence into public-health action.
